# High-flow nasal therapy versus non-invasive ventilation for AECOPD: navigating beyond a simple choice– are we asking the right questions??

**DOI:** 10.1186/s13613-025-01510-7

**Published:** 2025-07-02

**Authors:** Mauro Castro Sayat, Nicolás Colaianni-Alfonso, Adrián Gallardo, Luigi Vetrugno

**Affiliations:** 1 Respiratory Intermediate Care Unit, Juan A. Fernandez Hospital, Av. Cerviño 3356, Buenos Aires, C1425 Argentina; 2 Department of Kinesiology and Respiratory Care, “Sanatorio Clínica Modelo de Morón”, Morón, C1015 Argentina; 3 Department of Health Sciences, Kinesiology and Physiatry, National University of La Matanza, San Justo, B1754 Argentina; 4 Department of Emergency, Health Integrated Agency of Friuli Centrale, Udine, 33100 Italy; 5 Department of Medical, Oral and Biotechnological Sciences, University of Chieti-Pescara, Chieti, 66100 Italy

Dear Editor,

We read with great interest the recent meta-analysis by Qin et al. [1], which addresses a clinically significant question: the comparative efficacy of high-flow nasal cannula (HFNC) versus non-invasive ventilation (NIV) in patients with acute exacerbations of chronic obstructive pulmonary disease (AECOPD) complicated by hypercapnic respiratory failure. While this study offers valuable insights, we believe that its conclusion regarding the inferiority of HFNC compared to NIV warrants a more nuanced interpretation. The management of respiratory failure in AECOPD is inherently complex and requires an individualized approach that extends beyond binary treatment comparisons.

To begin with, the study’s dichotomous framework—contrasting HFNC with NIV as mutually exclusive strategies—does not fully reflect the dynamic and integrative nature of current clinical practice. Increasingly, clinicians adopt a personalized, adaptive model that includes the sequential or even concurrent use of both modalities [2, 3]. This evolving paradigm highlights the need for a broader, more multifactorial understanding of respiratory support strategies, rather than an oversimplified comparative evaluation. Moreover, while patient preference is a relevant consideration, it should not be allowed to obscure the multifaceted decision-making process that characterizes individualized care in these patients.

Importantly, the analysis does not account for key physiological parameters—such as baseline and post-intervention PaCO_2_ levels or diaphragmatic function—that have been shown to be significant predictors of HFNC success, nor does it consider composite scores like HACOR or ROX Index. Evidence suggests that the degree of hypercapnia and diaphragm performance are critical determinants in the efficacy of non-invasive respiratory support [3]. Furthermore, prolonged use of NIV prior to intubation has been associated with worse outcomes in patients with de novo hypoxemic respiratory failure, though this trend appears less pronounced in those with chronic lung disease [4]. In addition, recent propensity-matched studies have demonstrated that patients who respond favorably to HFNC often experience shorter durations of mechanical ventilation, decreased ICU and hospital length of stay, and reduced overall costs. Notably, patients who initially fail HFNC but are subsequently transitioned to NIV have shown comparable outcomes to those managed with NIV alone suggesting that a flexible, responsive approach to therapy does not compromise prognosis [5]. These critical aspects were not addressed in the current meta-analysis and merit further exploration, especially in the context of AECOPD.

Lastly, we acknowledge the authors’ transparency regarding the limitations of their analysis, particularly the small number of included studies. This limitation may significantly reduce the statistical power, affecting the external validity and hinder the detection of meaningful clinical differences between HFNC and NIV.

In summary, while Qin et al. make an important contribution to the discourse on respiratory support in AECOPD, a more comprehensive approach is essential—one that considers physiological predictors, optimal timing of interventions, and the integration of multiple therapeutic modalities. Addressing these dimensions will be crucial to refining management strategies and improving outcomes in this vulnerable patient population. We strongly encourage future research efforts to explore these underexamined but clinically significant questions.

## Data Availability

Not applicable.
